# Experimentally Induced Burns in Rats Treated with Innovative Polymeric Films Type Therapies

**DOI:** 10.3390/biomedicines11030852

**Published:** 2023-03-10

**Authors:** Oxana-Madalina Grosu, Oana-Maria Dragostin, Ioannis Gardikiotis, Carmen Lidia Chitescu, Elena Lacramioara Lisa, Alexandra-Simona Zamfir, Luminita Confederat, Ionut Dragostin, Maria Dragan, Catalina Daniela Stan, Carmen-Lacramioara Zamfir

**Affiliations:** 1 Department of Surgery I, Faculty of Medicine, “Grigore T. Popa” University of Medicine and Pharmacy, 16 Universitatii Street, 700115 Iasi, Romania; 2 Research Centre in the Medical-Pharmaceutical Field, Faculty of Medicine and Pharmacy, “Dunarea de Jos” University of Galati, 47 Domneasca Street, 800008 Galati, Romania; 3 Advanced Centre for Research-Development in Experimental Medicine, “Grigore T. Popa” University of Medicine and Pharmacy, 16 Universitatii Street, 700115 Iasi, Romania; 4 Medical Department III, Faculty of Medicine, University of Medicine and Pharmacy “Grigore T. Popa”, 16 Universitatii Street, 700115 Iasi, Romania; 5 Department of Biomedical Sciences, Faculty of Medical Bioengineering, University of Medicine and Pharmacy “Grigore T. Popa”, 16 Universitatii Street, 700115 Iasi, Romania; 6 Department of Pharmaceutical Science, Faculty of Pharmacy, “Grigore T. Popa” University of Medicine and Pharmacy, 16 Universităţii Street, 700115 Iaşi, Romania; 7 Department of Morpho-Functional Sciences I, Faculty of Medicine, “Grigore T. Popa” University of Medicine and Pharmacy, 16 University Street, 700115 Iasi, Romania

**Keywords:** burns, wound healing, oxytetracycline, polymeric films, chitosan

## Abstract

Considering that microbial resistance to antibiotics is becoming an increasingly widespread problem, burn management, which usually includes the use of topical antimicrobial dressings, is still facing difficulties regarding their efficiency to ensure rapid healing. In this context, the main objective of this research is to include new oxytetracycline derivatives in polymeric-film-type dressings for the treatment of wounds caused by experimentally induced burns in rats. The structural and physico-chemical properties of synthesized oxytetracycline derivatives and the corresponding membranes were analyzed by FT-IR and MS spectroscopy, swelling ability and biodegradation capacity. In vitro antimicrobial activity using Gram-positive and Gram-negative bacterial strains and pathogenic yeasts, along with an in vivo study of a burn wound model induced in Wistar rats, was also analyzed. The newly obtained polymeric films, namely chitosan-oxytetracycline derivative membranes, showed good antimicrobial activity noticed in the tested strains, a membrane swelling ratio (MSR) of up to 1578% in acidic conditions and a biodegradation rate of up to 15.7% on day 7 of testing, which are important required characteristics for the tissue regeneration process, after the production of a burn. The in vivo study proved that chitosan-derived oxytetracycline membranes showed also improved healing effects which contributes to supporting the idea of using them for the treatment of wounds caused by burns.

## 1. Introduction

Globally, burns are the fourth most frequent type of trauma. Up to 90% of burns occur in low- or middle-income nations, and the risk of burns tends to rise with poorer socioeconomic levels [1]. In burn units, general and local treatment remains challenging, and sepsis is the leading cause of death [2].

The local treatment of a burn depends on its degree. For a first-degree, minor burn, keeping it clean and moisturized is enough, the healing being very fast. In the case of partial-thickness burns, re-epithelialization is encouraged. Wounds are washed with soap and water, after which debridement of the loose skin and blisters is practiced [1]. Choosing the right dressing is very important, all the more so because currently there is no perfect dressing developed to allow complete healing without surgery or without changing the dressing. Ideally, one dressing should improve and speed up the healing process and also should prevent infection [3]. 

Classically, rolls of gauze are applied to soak up drainage after applying an antibiotic ointment with a non-adherent dressing on burn wounds [4]. Nowadays, there are special dressings for burn wound areas, such as active hydrogel, honey-based or chitin-based dressings. Active hydrogel dressings can be associated with agents with anesthetic, nutritional or anti-inflammatory properties, which can remove heat from the wound through convection and evaporation, and have wound-covering functions. Honey-based dressings use the antibacterial, anti-parasitic and analgesic properties of the honey, create a moist environment that maintains the burn surface’s integrity and form a bacterial barrier to prevent cross-contamination. Used as fibers, powders, granules, sponges, or composites with cotton or polyester, chitin and chitosan stimulate the wound healing process, maintain a sterile exudate environment and optimize healing conditions [3]. 

We can opt for a topical antimicrobial dressing, especially for its indications in preventing and treating burn infections, but then we have to change the dressing once or twice a day. Using silver sulfadiazine is to the detriment of the re-epithelialization process, so it can be used only for deeper burns. On the other hand, active silver ions have powerful bactericidal and antifungal proprieties, remaining a good alternative, but we need to stop it within a week because it causes rashes [1].

On the other hand, mafenide acetate can be used without dressings, but it can cause metabolic acidosis, so it is not indicated for large burn surfaces [2]. Another option is represented by “extended” or “closed” dressings, which stick to the wound, keep it wet and then fall off once the wound has healed. We can change them after 5 to 10 days. Excision and skin grafting are indicated in the case of wounds that need more than 2–3 weeks to heal because these wounds have a high chance to become hypertrophic scars and for full-thickness burns. As for the surgical procedure, the principles include the excision of the non-viable tissue, hemostasis and skin graft application harvested from a different anatomical location [1]. 

Moreover, in order to accelerate the healing process, we can harvest epithelial cells and spray them over the wound [1,5]. However, burn healing is a long, tedious and slow process, which needs dressings adapted to the new daily conditions, covering a long period of time. Considering that burns represent complex wounds that can lead to significant complications, they require an effective, well-adapted and prompt treatment. The problem that arises in the case of the modern treatment of burns consists in replacing a classic dressing with a wound dressing material impregnated with different therapeutics, such as antimicrobial or anti-inflammatory agents [6]. Unfortunately, the extensive prescription of antibiotics in conventional formulations has led to the appearance of microbial resistance, a fact that limits their effectiveness. Therefore, new strategies are needed to restore the antimicrobial activity of some classical antibiotics, as is the case with tetracyclines. Such strategies can be based on the synthesis or biosynthesis of new classes of drugs with unique antimicrobial properties but also on obtaining new formulations that can ensure the reduction of antibiotic consumption worldwide.

In addition, another important property of a dressing is ensuring hemostasis and tissue regeneration, which is why chitosan was chosen in our study as a biomaterial that promotes these properties [7]. To our knowledge, a wound dressing containing oxytetracycline, hydrocortisone and chitosan has not been investigated in acute and chronic wound models to date. Thus, any attempts to define a new therapeutic approach must work with the goal of ongoing improvement. Therefore, in our case, the aim of this study is to highlight the role of new chitosan-oxytetracycline derivative membranes in healing burn wounds, using an animal model. In order to fulfill the final goal of our work, a series of intermediate activities were carried out. We started with the chemical synthesis of oxytetracycline derivatives, obtaining polymeric membranes that contain these derivatives, together with a corticosteroid. We continued with the physico-chemical, spectral and in vitro biological characterization and the in vivo evaluation of polymer film dressings, based on an experimentally induced burn model in rats.

Currently, there is not a suitable perfect animal model that could reproduce wound healing in humans. The lack of such a perfect model is a consequence of a large number of limitations generated by the particularities of skin and burn wound healing in experimental animals [8]; at the same time, we must take into account the advantages of such experiments, which can offer the possibility to understand the complex pathophysiology of burn wounds and also use new therapeutic approaches.

## 2. Materials and Methods

### 2.1. Materials

All the reagents, chitosan medium molecular weight (CS MMW, 425 kDa, deacetylation degree of 85%), oxytetracycline hydrochloride (OXI), hydrocortisone, aromatic benzaldehydes (2-NO**_2_**-benzaldehyde, benzaldehyde, 4-Br-benzaldehyde, 4-OH-benzaldehyde), acetic acid, sodium hydroxide, sodium tripolyphosphate (Na-TPP) and organic solvents (p.a.), were purchased from Sigma–Aldrich (Romania). All the other materials were of analytical reagent grade and were used as received.

### 2.2. Description of the Chemical Synthesis Protocol

Oxytetracycline derivatives were obtained following condensation reactions between pure oxytetracycline and four types of aromatic benzaldehydes, in a 1:1 molar ratio. Thus, oxytetracycline (0.001 moles) was dissolved by heating in 50 mL of absolute ethyl alcohol, and the amount of each aromatic benzaldehyde (0.001 moles) was added to the obtained solution to carry out four different reactions. To each reaction mixture, 2 mL of glacial acetic acid was added, all being kept on a magnetic stirrer, in a water bath under reflux, for 8 h. The resulting precipitate at the end of the reaction time was separated by filtration, washed with distilled water, dried at room temperature and recrystallized from suitable solvents. The general synthesis scheme was developed by adapting some condensation methods of other new compounds with similar structure [9,10].

### 2.3. Physico-Chemical and Spectral Characterization

After obtaining the new oxytetracycline derivatives, their physico-chemical characterizations were carried out, in which the following were addressed: the chemical formula of the compounds, the relative molecular mass, the melting point and their yields. In addition, the chemical structure of the hydrazones was confirmed by IR and MS spectroscopy. 

Infrared spectra were recorded using an Agilent Cary 630 FTIR Spectrometer (USA), after 32 scans on a scale of 4000–500 cm^−1^, with a spectral resolution of 4 cm^−1^. Interpretation of the results was performed using Agilent MicoLab PC software, Automated IQ/OQ, 21 CFR Part 11 compliant, Resolutions Pro, for advanced data analysis.

High-resolution mass spectrometry (HRMS-MS) analysis was also carried out for structural confirmation of the synthesized compounds, according to the methods applied in a similar way, to other synthetic compounds [11,12].

#### 2.3.1. LC Parameters

A Thermo Scientific Dionex Ultimate 3000 UHPLC (Thermo Fisher Scientific, Waltham, MA, USA) was used for analysis. Chromatographic separation was performed with an Accucore U-HPLC Column C18 (150 × 2.1 mm, 2.6 µm) (Thermo Scientific) using a flow rate of 0.4 mL/min. The mobile phase consisted of water with formic acid, 250 µL/L (A), and methanol with formic acid, 250 µL/L (B). A 15 min gradient was used. The step gradient was as follows: 0–1 min 100% A; 1–2.5 min linear increase to 40% B; 2.5–10 linear increase to 100% B and hold 3 min; 13–13.2 decrease to 0% B; 13.2–15 min 100% A. Injection volume was set at 20 μL. HESI (heated electrospray) ion source was used for the ionization in positive mode. The HESI parameters were optimized as follows: sheath gas flow rate 40 unit; aux. gas unit flow rate 10; capillary temperature 250 °C; aux. gas heater temperature 300 °C; spray voltage 2800 V; S lens RF level 50.

#### 2.3.2. MS Parameters

Detection of the target compounds was performed using a Q-Exactive mass spectrometer (Thermo Fisher Scientific, USA). The analytical approach included a full MS screening at a resolving power of 70,000 FWHM at m/z 200 followed by MS-MS analysis in MRM (multiple reaction monitoring) mode at 35,000 FWHM. Collision energy was consecutively increased from 20 to 30, 35 and 45 eV. Precursor ions are filtered by the quadrupole which operates at an isolation window of m/z 2.

Data were evaluated with the Quan/Qual Browser Xcalibur 2.3 (Thermo Fisher). The mass tolerance window was set to 5 ppm for the two analysis modes. Confirmatory analysis was based on accurate mass measurements and pattern recognition of the product ions comparing MS/MS data generated by HighChem Mass Frontier 7.0 Xcalibur 4.1 software. 

### 2.4. Obtaining of Chitosan-Oxytetracycline Membranes

**Figure 1:**
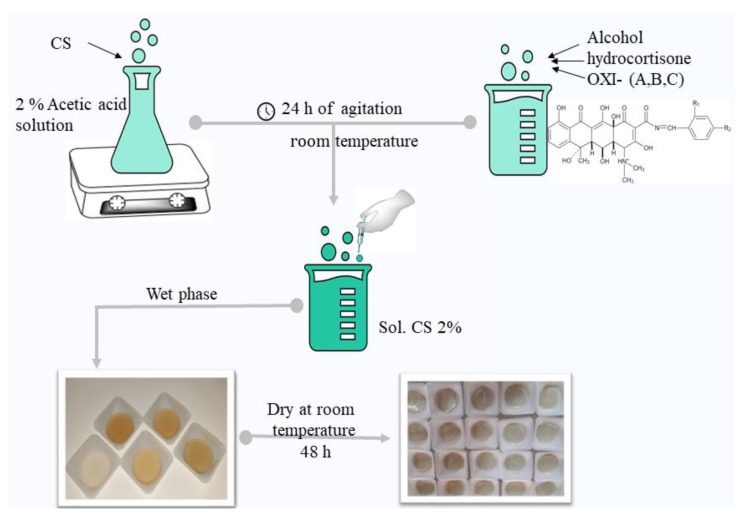
The stages of obtaining CS-OXI derivative film type membranes.

In order to obtain new polymeric-film-type therapies, chitosan was first dissolved in 2% acetic acid, for a final concentration of 2% (*w*/*v*), by stirring on a magnetic stirrer at room temperature, for 24 h. The new oxytetracycline derivatives, obtained according to the previously described procedure, were dissolved in a minimal amount of 5 mL absolute ethyl alcohol, while the obtained hydrocortisone powder was added to the solution, for a 0.1% (*w*/*v*) final concentration. This alcoholic solution was added dropwise to the initial 2% chitosan solution in acetic acid. Further, 15 mL was taken from the final solutions and placed on 10/10 cm polyethylene plates, in order to evaporate the solvent at room temperature (Figure 1). The working protocol was established according to a study previously carried out [13]. Polymer films were made using each new oxytetracycline derivative (OXI A→C, excluding OXI-D for reasons of solubility). Moreover, pure oxytetracycline and plain chitosan were used for further elucidation of increased therapeutic effect. Finally, the membranes were cross-linked with 2% NaTPP (sodium tripolyphosphate) solution.

### 2.5. Characterization of Chitosan-Oxytetracycline Membranes

#### 2.5.1. In Vitro Swelling Ratio

According to the literature [14], the therapeutic efficacy is also significantly influenced by the ability of the polymer membrane to retain water, this being tested by determining the degree of swelling at equilibrium. 

The swelling behavior of chitosan-oxytetracycline-derivative cross-linked films was studied in three different pH environments (acidic, neutral and basic) to mimic the pH of wounds in burns of various etiologies. These environments are represented by acetate buffer (pH 5), distilled water (pH 7) and ammonia buffer (pH 10). The films were cut into small pieces of known weight (Wd) and then placed on polyethylene plates in 10 mL of distilled water and left at room temperature. At 10 min intervals, the samples were removed, excess water was removed by gentle dabbing using filter paper, and then the samples were weighed (Ww) on an analytical balance and re-introduced into water, repeating the operation until constant weight of the samples. The same procedure as described above for distilled water was also followed for the acetate and ammonia buffer.

The membrane swelling ratio (MSR) of chitosan membranes at different time intervals was calculated according to the following formula:

(1) $$MSR\left(\%\right)=\frac{w_{w}-w_{d}}{w_{d}}\times 100$$

where *W_w_* = dry sample weight, and *W_d_* = wet sample weight at different time intervals [15].

#### 2.5.2. In Vitro Biodegradation

Polymeric films of chitosan-oxytetracycline derivatives, previously cross-linked with TPP and of known weight, were placed in PBS solution for about 60 min until the swelling equilibrium of the membranes was reached and then weighed to find out the wet weight before incubation (W_0_). After that, the PBS solution was changed to PBS solution containing lysozyme 10,000 IU/ ml. The samples thus immersed in the PBS solution with lysozyme were incubated at 37 °C for 7 days. At different period times (1, 4 and 7 days), film samples were taken out of solution and weighed (*W_w_*) [16].

The degree of in vitro biodegradation of the polymer films was calculated according to the following formula:

(2) $$D(\%)=\frac{W_{0}-W_{x}}{W_{0}}\times 100$$

where *W*_0_ = wet weight of films before incubation, and *W_x_* = wet weight of films after incubation.

### 2.6. Biological Evaluation

#### 2.6.1. Assessment of Antimicrobial Activity

The new oxytetracycline derivatives obtained through chemical synthesis (OXI-A, OXI-B, OXI-C and OXI-D) were evaluated concerning their antimicrobial activity against Gram-positive bacterial strains (*Staphylococcus aureus* ATCC 25923), Gram-negative bacterial strains (*Escherichia coli* ATCC 25922, *Pseudomonas aeruginosa* ATCC 27853) and pathogenic yeasts (*Candida albicans* ATCC 90028). The bacterial and fungal suspensions were obtained by dispersing a small amount of each microbial culture in sterile NaCl 0.9% until a turbidity equivalent to McFarland standard no. 0.5 (106 CFU/mL). 

The culture medium used for antimicrobial susceptibility tests was Mueller Hinton agar for antibacterial tests and Sabouraud agar for antifungal tests. The culture medium was spread on sterile Petri plates, in volume of 25 mL/Petri plate. 

For testing the antimicrobial activity of synthetic derivatives, parent oxytetracycline and the four new derivatives (OXI-A, OXI-B, OXI-C, OXI-D) were dissolved in concentration of 4.7 mg/mL. 

Disc-diffusion method: Antimicrobial tests were performed according to CLSI specifications [17,18]. The bacterial and fungal suspensions, prepared as described above, were inoculated on the surface of the culture medium spread on Petri plates. Sterile stainless steel cylinders (5 mm internal diameter and 10 mm height) were applied on the agar surface on Petri plates, and 100 µL of each compound tested was added into cylinders, the final concentration tested being 0.47 mg. Commercial discs containing tetracycline (30 µg/disc), ciprofloxacin (5 µg/disc), fluconazole (25 µg/disc) and voriconazole (1 µg/disc) were used as positive control. DMSO was used as negative control. The plates were incubated at 37 °C for 24 h (antibacterial activity) and at 24 °C for 48 h (antifungal activity). After the incubation, the diameters of the inhibition area (mm), including disc size, were measured. 

Broth micro-dilution method: Briefly, 100 µL Mueller Hinton broth was added to a 96-well microplate, and two-fold dilutions of the tested compounds were prepared from a stock solution of 4.7 mg/mL. Then, a small volume of bacterial and fungal strain suspension, 106 CFU/mL (McFarland standard no. 0.5), was inoculated into the wells, and the microplates were incubated at 37 °C for 24 h (bacterial strains) and at 24 °C for 48 h (fungal strains). The MIC value was established as the lowest concentration, which determined the visual inhibition of the strain’s growth. For MBC/MFC determination, a small volume from each plate with visually complete inhibition was transferred to a solid medium plate and incubated for 24 h at 37 °C (bacterial strains) and at 24 °C for 48 h (fungal strains). The MBC/MFC was considered the lowest concentration, which killed 99.9% of the tested strains.

#### 2.6.2. In Vivo Study

The present study received the approval of the Ethical Committee of Grigore T. Popa University of Medicine (ID OMG/12.03.2019), while the use and treatment of laboratory animals were carried out in accordance with international ethical standards.

Male Wistar rats aged 12 weeks, weighing 220–250 g, were used. The animals were organized into 6 working groups of 7 animals per group (untreated control and those that received local treatment with different types of membranes: CS, CS-OXI, CS-OXI-A, CS-OXI-B, CS-OXI-C) and kept in temperature-controlled cages with unlimited access to food and water and were kept on a 12 h light/dark cycle. The rats were housed individually throughout the experiment to prevent aggressive behaviors that are known to cause pain and could have further injured or killed the rats.

In order to conduct the experiment, the animals were given isoflurane gas inhalation mixed with regular air (5% for induction and 2% for maintenance), which rendered them unconscious. The dorsal skin was then shaved using a razor then washed using a solution of benzalkonium chloride (50%, 9.85 mg) and chlorhexidine digluconate (20%, 10.65 mg each). In order to produce burn lesions with the potential of them turning into hypertrophic scars, we used a circular filter paper with a diameter of 2 cm soaked in a 3 N sodium hydroxide aqueous solution. A deepidermization was performed with the razor after shaving the hair. The filter sheet was applied for 1 min, directly at the level of the previously denuded dermis, after administering these mechanical and chemical injuries to the skin. 

The control lesion was on the left dorsal thorax, with nothing else being applied. On the right dorsal thorax, after creating the lesion, we applied the new polymer films previously described (CS, CS-OXI, CS-OXI-A, CS-OXI-B and CS-OXI-C). After soaking the biofilm in physiological saline, it was applied to the level of the burn injury and was secured with surgical staples.

Immediately following the burn, an intra-peritoneal injection of 40 mg/kg tramadol was administered to the animals to treat their pain. In order to achieve proper tightness without being circular and at risk of chest compression, all lesions were bandaged with sterile dressings placed directly to the wound and maintained using X-banded adhesive tape. The applied substances as biofilms were removed after three days. The welfare of the animals was regularly checked. When needed, the dressing was changed (otherwise, every follow-up day). The working protocol was adapted from the specialized literature, from similar studies carried out on laboratory animals [19,20,21,22]. 

The wounds were photographed on days 5 and 14 before sacrification. The mean area of the wounds was measured based on the pictures. The effect of the wound dressing on wound closure rate was measured according to the following formula [23]:

(3) $$Wound\;closure(\%)=\frac{Initial\;wound\;area\;-\;wound\;area\;(day\;5\;and\;14)}{Initial\;wound\;area}\times 100$$

The animals were euthanized on days 5 and 14, using a hyperdose of anesthesia, and the entire-thickness wound tissue with a minimum of 0.5 cm of normal tissue borders was then collected for histological research. The samples were then specifically used for paraffin embedding and staining with hematoxylin–eosin (HE). A microscopic exam was performed on Nikon Eclipse microscope. 

### 2.7. Statistical Analysis

All tests were performed in triplicate, and the data are expressed as means ± SD. The statistical software package Stat View was used for data analysis of biological assays. Experimental results were analyzed by 3 (groups) × 3 (time sample points) repeated-measures ANOVA and Fisher’s post hoc test to compare the burn surface area between control and tested groups at days 5 and 14. The criterion for significance was *p* < 0.05.

## 3. Results

### 3.1. Chemistry

**Figure 2:**
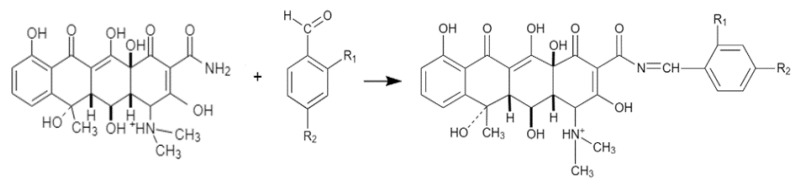
Synthesis of oxytetracycline derivatives. OXI-A (R_1_ = –NO_2_; R_2_ = –H); OXI-B (R_1_ = –H; R_2_ = –H); OXI-C (R_1_ = –H; R_2_ = –Br); OXI-D (R_1_ = –H; R_2_ = –OH).

In order to obtain oxytetracycline derivatives, several benzaldehydes were reacted with the parent compound (OXI) in dry acetone in the presence of anhydrous potassium carbonate to obtain four *N-* substituted oxytetracycline derivatives (Figure 2). The idea of binding an aromatic nucleus (substituted or not) to the oxytetracycline structure, by means of an azomethine group, aims to extend the duration of action of the new molecules (through their new physico-chemical properties, high molecular mass and increased lipophilicity degree), as well as to improve the antimicrobial spectrum, due to the newly introduced benzaldehydes, known for their antimicrobial properties [24]. 

### 3.2. Characterization of Oxytetracycline Derivatives

#### 3.2.1. Spectral Data

**Figure 3:**
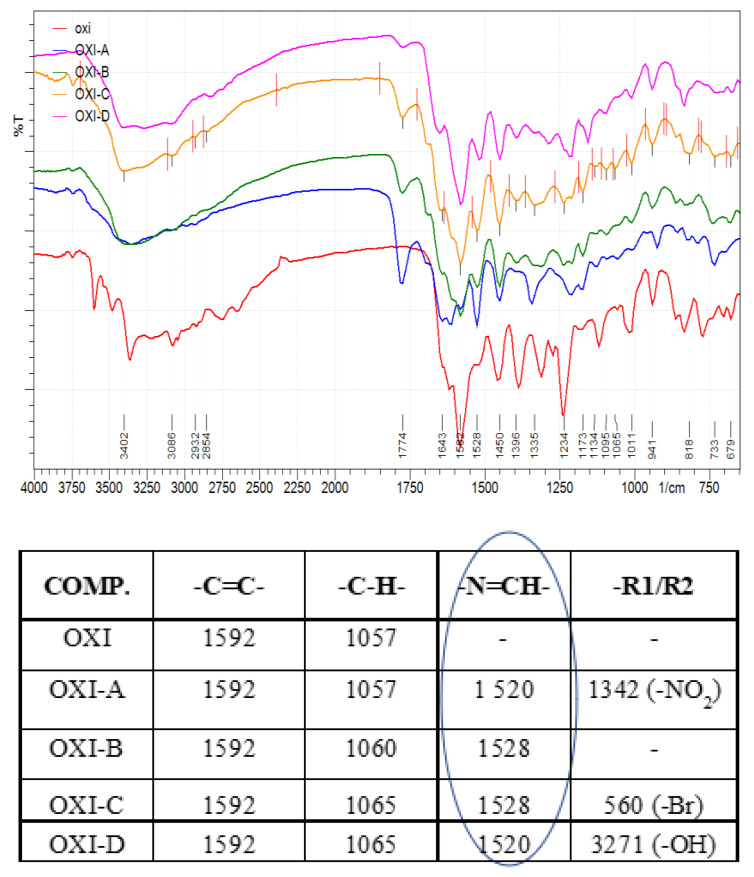
IR spectra of oxytetracycline derivatives and the peaks corresponding to functional groups.

In the IR spectrum of the four hydrazones, the azomethine functional group (-N=CH-) was identified, by the appearance of the high-intensity absorption band in the 1520–1528 cm^−1^ range (Figure 3). The appearance of this band constituted an argument for the condensation reaction that took place between oxytetracycline and the aromatic aldehydes selected for this study (2-NO_2_-benzaldehyde, benzaldehyde, 4-Br-benzaldehyde, 4-OH-benzaldehyde).

Moreover, apart from the azomethine functional group, all the other elements specific to the aromatic nucleus in the structure of oxytetracycline and aromatic benzaldehydes were identified. This is found in the spectrum both through the bands of average intensity in the region 1057–1065 cm^−1^, due to the stretching vibrations of the -C-H- bond, and through the bands of average intensity at 1592 cm^−1^, attributed to the stretching vibrations of the -C-C- bond. 

At the same time, the aromatic component used in the condensation reaction was identified by the substituent in position no. 2 in the case of the OXI-A derivative and the substituent in position no. 4 in the case of the OXI-C and OXI-D derivatives. Thus, in the case of using 2-NO_2_-benzaldehyde, the presence of the -NO_2_ functional group (for OXI-A compound) was confirmed by the antisymmetric valence vibrations from 1342 cm^−1^, while in the case of using 4-Br-benzaldehyde, the presence of brom halogen (for OXI-C compound) was confirmed at 560 cm^−1^. In the case of the OXI-D compound, the presence of the -OH group in the *para* position of the derivative was identified at 3271 cm^−1^.

**Figure 4:**
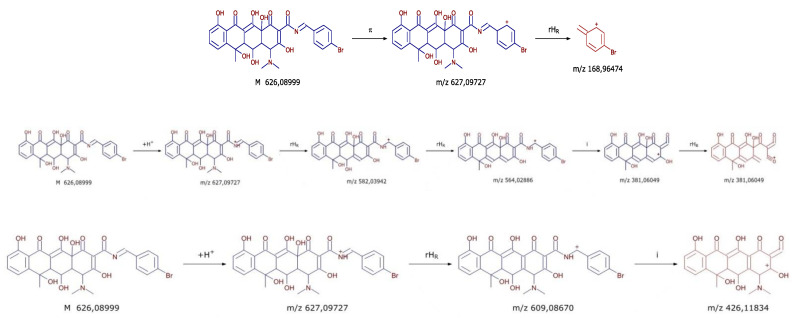

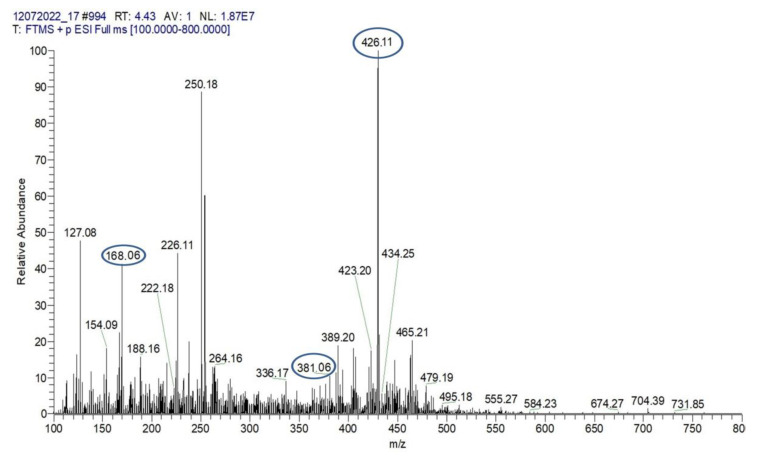
Fragmentation pattern generated by Mass FrontierTM and MS2 spectra for the derivate OXI-C.

In the MS spectrum case, the fragment ions m/z 426.1183, 381.0604 and 201.0546 are common for all compounds and are characteristic of the same parent compound (oxytetracycline). The additional benzoic ring with the specific radical of each derivate was identified, confirming the chemical structure (in bold in Table 1). As an example in Figure 4, the fragmentation pattern identified and compared to the fragmentation patterns generated by the Mass Frontier software of the compound OXI-C is shown.

#### 3.2.2. Physico-Chemical Characterization

The new oxytetracycline derivatives were presented in the form of crystalline powders, with colors that varied from yellow to brown, with a characteristic smell. All the compounds obtained showed a fixed melting point, which indicated that they were obtained and separated in a pure form. Following the optimization of the synthesis method, the oxytetracycline condensation compounds were obtained in very good yields, which varied between 69.23% and 83.85% (according to Table 2).

### 3.3. Characterization of Chitosan-Oxytetracycline Membranes

Chitosan derivative membranes were prepared using sodium tripoly-phosphate (TPP) as a crosslinking agent. It is known that non-cross-linked chitosan membranes have poor chemical stability and will dissolve in an acid environment and therefore need to be cross-linked. Free amine groups of chitosan derivatives are protonated in acid conditions and consequently are positively charged. In the presence of TPP, a network is produced based on electrostatic interactions between the negatively charged crosslinking agent and positively charged glucosamine chains [25].

#### 3.3.1. In Vitro Swelling Ratio

**Figure 5:**
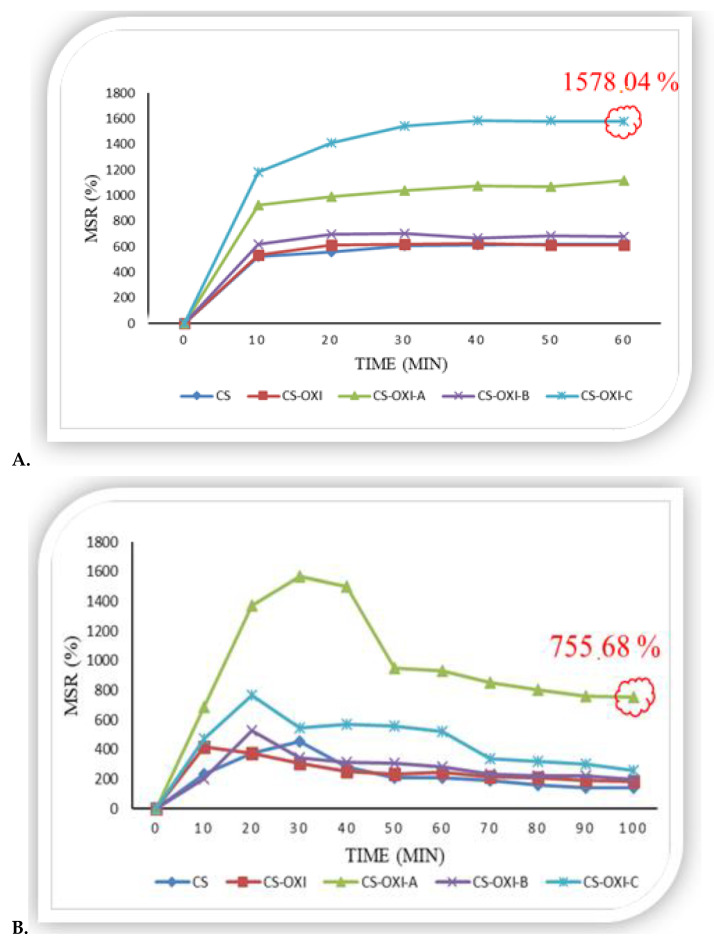

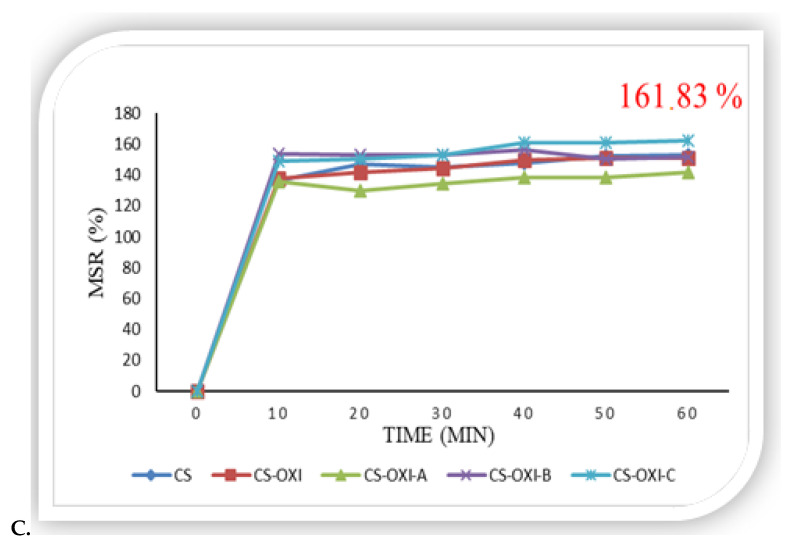
Membrane Swelling Ratio of polymer films at 5 (**A**), 7 (**B**) and 10 pH (**C**).

For acetate buffer (pH 5—acid medium), the swelling equilibrium of the CS films was reached after 60 min, the swelling ratio recorded being MSR = 617.8%. The polymer films containing oxytetracycline derivatives (CS-OXI-A, CS-OXI-B and CS-OXI-C) absorbed an increased amount of acetate buffer, already in the first 10 min of the experiment, with a visibly increased swelling ratio at equilibrium compared to the pure CS membrane, the highest percentage being registered in the case of CS-OXI-C with the MSR value = 1578.04%, after 50 min (Figure 5A). 

For distilled water (pH 7—neutral medium), the swelling equilibrium of the CS film was reached after 100 min, the swelling ratio recorded being MSR = 143.83%. The CS-OXI-B and CS-OXI-C polymer films showed a slightly increased equilibrium swelling ratio compared to the CS film. The highest percentage was highlighted in the case of the CS-OXI-A film with MSR = 755.68%, after 100 min (Figure 5B).

In the case of ammonia buffer (pH 10—basic medium), the swelling equilibrium for both the CS film and the polymer films with oxytetracycline derivatives (CS-OXI-A, CS-OXI-B and CS-OXI-C) was reached after a time interval of 60 min, and the percentages of the three type of films were similar to that of CS, which was about MSR = 150% (Figure 5C).

#### 3.3.2. In Vitro Biodegradation

The biodegradation of chitosan polymer films derived from oxytetracycline was studied using phosphate-buffered saline (PBS pH 7.4) to which lysozyme was added. 

**Figure 6:**
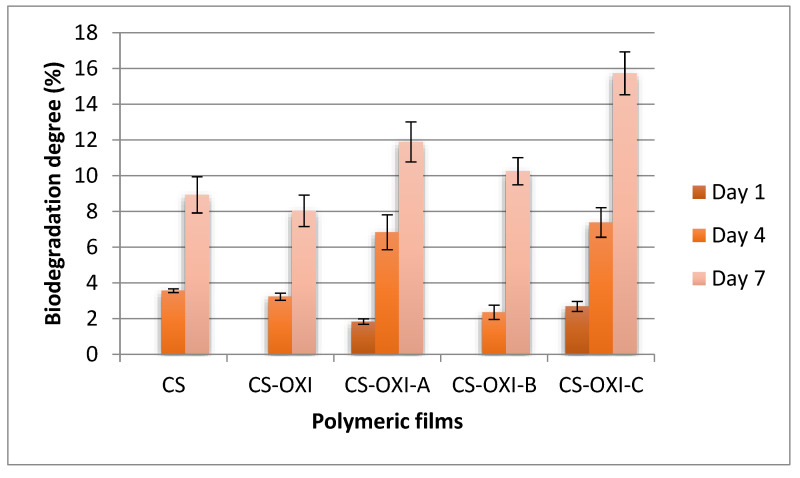
Biodegradation degree of polymeric films.

On the first and fourth days after the start of the in vitro biodegradation experiment, the oxytetracycline-derivative CS films were biodegraded at a percentage approximately equal to that of the CS film. At the end of the test, all films containing oxytetracycline derivatives were biodegraded at a higher ratio compared with CS membrane, for which the percentage was D% = 8.92%. The highest biodegradation rate was recorded in the case of CS-OXI-C with D% = 15.72% on the seventh day of the experiment (Figure 6).

### 3.4. Biological Evaluation

#### 3.4.1. Assessment of Antimicrobial Activity

Disc-diffusion method: The antimicrobial activity and diameters of the inhibition area corresponding to the tested compounds are shown in Table 3. 

Broth micro-dilution method: The minimum inhibitory concentration (MIC) and the minimum bactericidal/fungicidal conc. (MBC/MFC) values are presented in Table 4. 

From the obtained results, OXI-B proved to be more active than the parent compound (obtained by binding with unsubstituted benzaldehyde), the CMB being, in the case of *E. coli*, two times lower (0.073 vs. 0.14 mg/mL). For the rest of the compounds, antimicrobial activity remained generally unchanged or was even slightly reduced, as is the case with binding with 2-NO_2_ benzaldehyde (in OXI-A’s case).

#### 3.4.2. In Vivo Study: Wound Healing Assay

**Figure 7:**
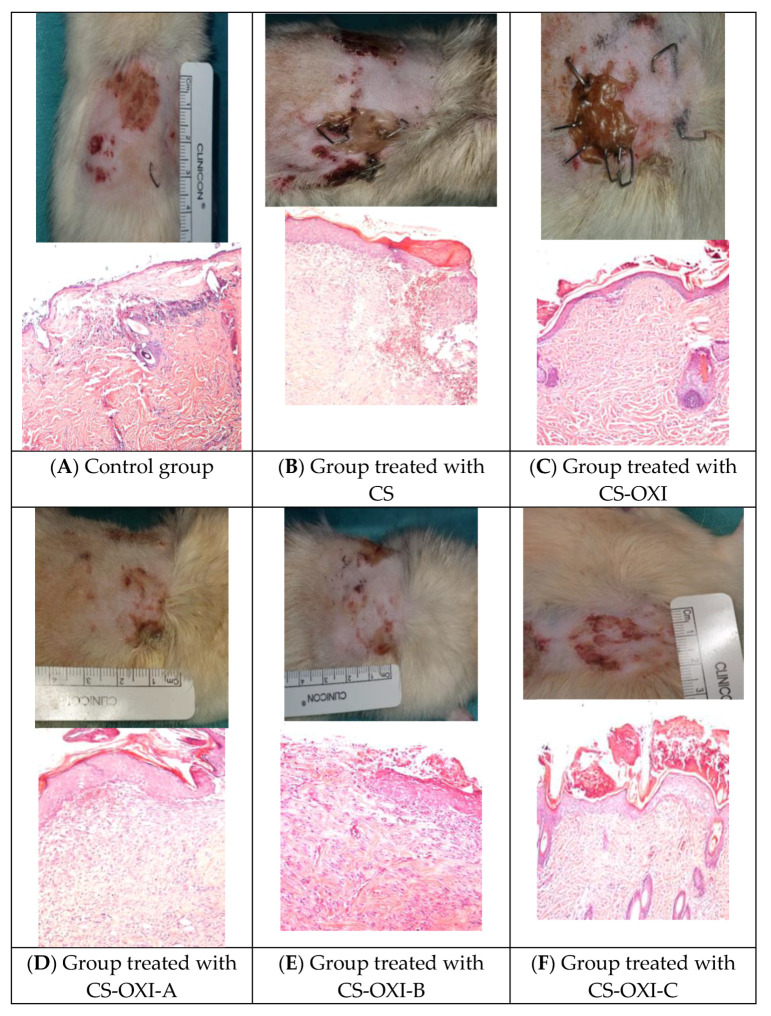
Microscopic and macroscopic images of burn wound at 5 days.

Five days after the burn, the most significant histopathological changes could be highlighted and revealed: for control (A), massive necrosis with an inflammatory infiltrate and hemorrhagic dermic areas were observed, while for pure chitosan (B), necrosis was also present, without an inflammatory infiltrate, but severe dermic hemorrhage could be observed; for the CS-OXI-treated group (C), reduced necrosis and inflammation were recorded; for the CS-OXI-A-treated group (D), necrosis, incipient inflammation and edema were observed; for the CS-OXI-B-treated group (E), severe necrosis and an abundant hyperemic area were present; and for the CS-OXI-C-treated group (F), necrosis, reduced fibrin deposition and disorganized collagen in the dermis were observed (Figure 7).

**Figure 8:**
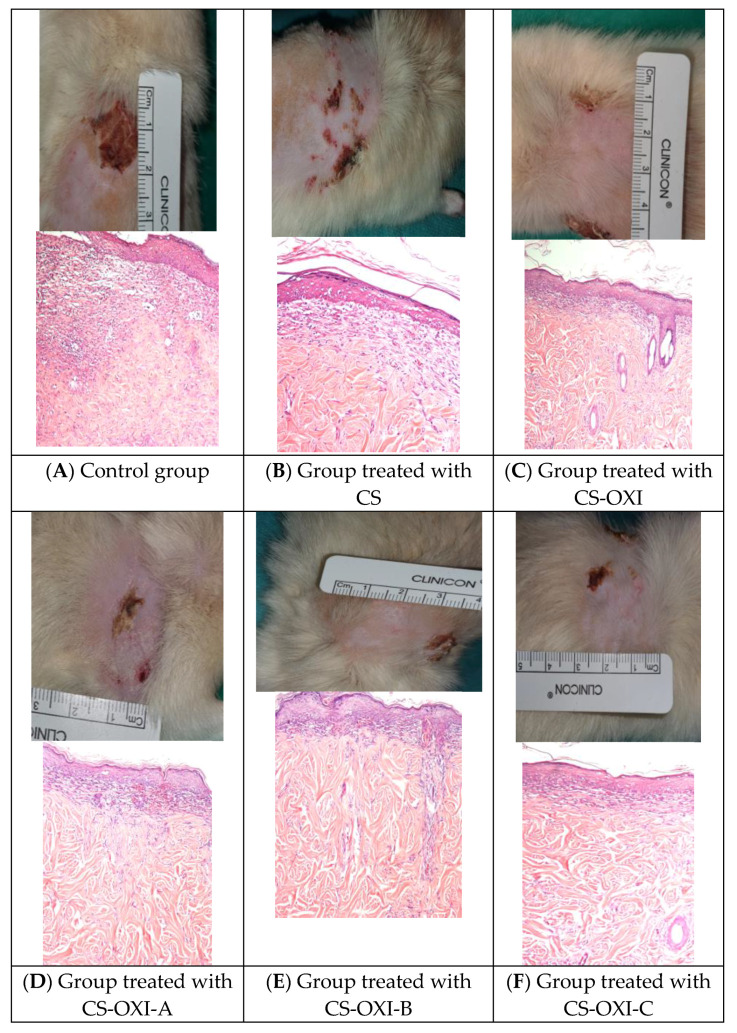
Microscopic and macroscopic images of burn wound at 14 days. (**A**) Control group; (**B**) Group treated with CS; (**C**) Group treated with; CS-OXI; (**D**) Group treated with CS-OXI-A; (**E**) Group treated with CS-OXI-B; (**F**) Group treated with CS-OXI-C.

Fourteen days after the burn, the following histopathological changes could be highlighted after the microscopic exam: for control (**A**), re-epithelialization at the edges of the wound, with a focal inflammatory infiltrate in the dermis, was observed while for pure chitosan (**B**), complete re-epithelialization and dermic remodeling areas could be observed; for the CS-OXI-treated group (**C**), re-epithelialization with diffuse inflammatory cells in dermic subjacent areas and the preservation of skin appendages were recorded; for the CS-OXI-A-treated group (**D**), a newly formed epithelium, with inflammatory infiltrate clearly organized collagen bundles in the profound dermis were observed; for the CS-OXI-B-treated group (**E**), re-epithelialization, dispersed inflammatory cells in the superficial dermis and angiogenesis were observed; and for the CS-OXI-C-treated group (**F**), complete re-epithelialization and collagen bundles normally configurated in the dermis could be observed (Figure 8).

**Figure 9:**
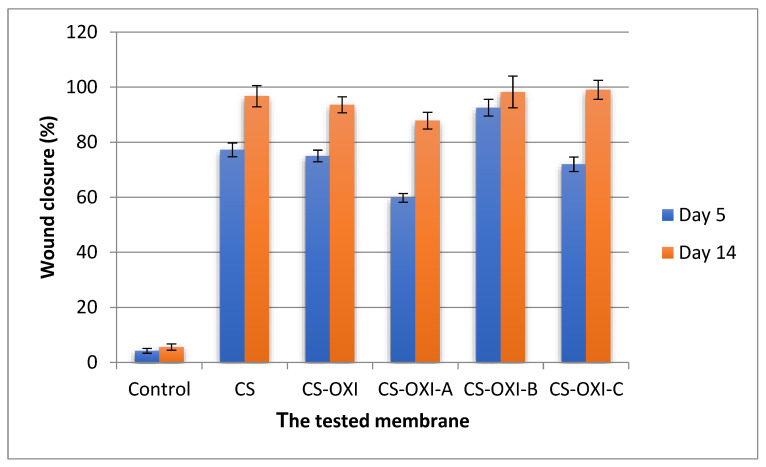
The wound closure (%) at the two different periods of time (on days 5 and 14).

The macroscopic exam of burns revealed the process of healing and the size of burns reported at two different moments of the experiment. The wound closure percentage of burn surface areas at different days during the recovery phase is shown in Figure 9. In correlation with the in vitro antimicrobial activity, where the OXI-B compound proved to be the most active, in the in vivo test, the CS-OXI-B membrane determined (from the fifth day) the greater reduction of the burn-affected surface area. On the other hand, the chitosan film also caused a significant reduction in the surface area affected by the burn, compared to the untreated control group (due to its intrinsic cicatrizing properties [15]), the effect being sustained by the presence of oxytetracycline derivatives in the membrane structure.

## 4. Discussion

The studies carried out so far on the structure of tetracyclines led to obtaining more active compounds, by introducing different substituents into the side chain, although the main condensed tetracyclic nucleus remains unchanged. This basic structure is important for antimicrobial activity, offering at the same time the ability to form complexes with bivalent metals (such as Mg^2+^, Cu^2+^, Ca^2+^), the resulting complexes having a stronger inhibitory effect [26]. For the antibiotic effect, a complex formed with magnesium ions inside bacterial cells is responsible, resulting in the inhibition of bacterial growth [27]. Thus, in our case, even the new compounds will have this property as long as the tetracyclic structure remains unchanged. In addition, the structural modification of oxytetracycline has, in some of the cases (OXI-B and OXI-C), a positive influence on the antimicrobial activity, both through the aromatic nucleus introduced into the structure of the new compounds and through the azomethine group, known for its impact on this effect [10]. Oxytetracycline inhibits cell growth by inhibiting translation [28], a mechanism that can be also considered for the derivatives obtained in our study, the main structure being kept in these cases as well.

The structural modification of some tetracyclines was also taken into account in the past by other researchers, who observed that the increase in the potency of the compounds resulting from the synthesis is closely related to the increase in the newly introduced radical at the carboxamide residue in the C2 position of the respective tetracycline [29]. Similarly, Sriram D. et al., 2007, modified the structure of oxytetracycline by substituting the group mentioned above with the rest of some fluoroquinolones, which led to the increase in the volume of the obtained compounds and to the intensification of the antimicrobial activity, the possible explanation being the exercise of a dual mechanism of action, by inhibiting both protein and DNA synthesis [29].

Numerous studies approach the subject of burn wounds—complex injuries that trigger multiple local and systemic reactions, diverse complications and long-term consequences. Special consideration is given to their management, especially to burn treatment, hence the continuous debate on the most efficient therapeutic approach. In our study, an animal model was used to observe experimentally induced burns and their healing process, related to new chitosan-derived oxytetracycline membranes evaluated for their wound healing properties vs. parent chitosan (CS) as a reference, which is well-known for its biocompatible and healing properties [15].

The exudate is generated during the wound healing process as part of the inflammatory response and is one of the components of the reparative process. It has a complex composition that includes glucose, leukocytes, cytokines, lysozyme and many other components. Lysozyme is an enzyme that is present in exudate, tears, nasal secretions, saliva, urine and other body fluids. The antimicrobial activity of this enzyme is related to the ability to hydrolyze the glycosidic bonds present in the peptidoglycans of the cell wall of Gram-positive bacteria [30]. One of its roles is to cleave the glycosidic bonds between the polysaccharide units in the polymer and to degrade chitosan into oligosaccharides [31]. It has been demonstrated that the activity of lysozyme in the degradation of chitosan decreases with the increase in its deacetylation degree [32]. In the present study, the biodegradation increases with the increase in the mass of the oxytetracycline derivative included in the polymer membrane, so that the highest percentages in the biodegradation test were recorded in the OXI-A and OXI-C derivative cases.

As a rule, polysaccharides have a high affinity for water, due to the large number of hydrophilic groups in the molecule, which can be easily hydrated [33]. The swelling degree of polymer films represents a very important test in the present research, influencing the absorption of exudates produced during the wound healing process. According to the literature, the swelling degree is an essential parameter in the active substance release from the polymeric matrix, having a significant effect on the bioavailability of the drug [34]. In this regard, the absorption capacity of new chitosan-derived oxytetracycline membranes was tested in different pH environments (pH 5, pH 7 and pH 10), using buffer acetate, distilled water and ammonia buffer, to closely mimic the pH of burn wounds of various etiologies and/or various healing stages [35]. According to the obtained results, the highest swelling capacity of the polymeric membranes was demonstrated at acidic pH.

In the case of burns, the skin is destroyed, and with its destruction, the immunological and bacteriological barrier also disappears, which makes the burned wound a favorable culture environment for the development of bacteria. This role of protection against microorganisms can be relatively easily replaced by a dressing, in the case of small burns. The dressing for minor burns has the following purposes: the absorption of exudate, protection and isolation of the wound from the environment and reduction of the pain to ensure the comfort of the patient [36]. Thus, through this research, we proposed to create an innovative dressing in which the dressing material itself has therapeutic properties, bringing a benefit by loading it with antimicrobial agents, such as the new oxytetracycline derivatives, and with an anti-inflammatory agent, such as hydrocortisone.

In our in vivo experiment, the burn degree was evaluated by the skin wound punch-biopsy staining protocol, while intermediate burn wounds were the result of sodium hydroxide aqueous solution exposure. New chitosan-derived oxytetracycline membranes were evaluated for their wound healing properties vs. chitosan (CS) as a reference. The polymeric membranes were fixed on top of the burn wound, while the burn wound healing was evaluated at 5 and 14 days after the start of the experiment. 

The initial affected surface area (on day 0) in all analyzed groups did not differ from one group to another, showing the effectiveness of the selected methods. The affected surfaces in rat groups treated with chitosan and chitosan-derived oxytetracycline membranes were significantly smaller than the untreated control group (*p* < 0.01) at both intervals of 5 and 14 days. All the dressing materials were more effective for wound healing than the control group (without treatment), CS+OXI C membranes having the best healing effect among all the groups and also having the highest MSR percentage, under the conditions of an acid pH.

The microscopic evaluation at 5 days post wounding pointed out that the evolution of the healing process was different, epithelial destruction, abundant necrosis and inflammatory infiltrates being observed in A, B and E, while in C, D and F, the restoration of epithelial tissue and the progression of healing were more visible. At the end of the experiment, 14 days post wounding, the epithelial regeneration was present in all the examined groups, but there were differences among groups regarding the healing process. Whereas the control group (A) presented an incomplete epithelial repair, with granular tissue and dispersed inflammatory cells, groups C, D and E presented complete epidermal regeneration, reduced granular tissue and a significant deposition of collagen. In groups B and F, the epithelial repair was complete, restoring the epidermal integrity, its layers are clearly well delimited, and the underlying dermis embeds bundles of collagen. 

Overall, this research investigated new types of dressings to overcome the disadvantages of conventional forms of antibiotic administration. Being non-conventional pharmaceutical forms, dressings based on polymers with active substances can be considered safe and efficient transport systems by ensuring their solubility in the specific environments of burn wounds [37]. Although initially burn wounds are considered free of microbial strains, contamination with pathogenic agents, such as Gram-positive and Gram-negative bacteria, takes place in a short time. For this reason, Tort S. et al. [23] have included in their study the use of doxycycline to obtain nanofiber dressings based on polymers such as chitosan and collagen which promoted the healing of wounds and stopping their chronicization. Electrospinning is a technique that ensures the production of high-performance dressings, facilitating the obtaining of structures similar to that of the extracellular matrix, which favors the healing process. Moreover, this strategy has been adopted by Castro K.C. et al. [38] for the production of dressings with a porous structure based on hyaluronic acid that improves homeostasis, gas permeability, exudate absorption, adhesion, migration and cell proliferation.

Recently, the solvent cast method has been also used by a group of researchers [39] to obtain antibacterial wound dressing candidate films, although their ability for tissue regeneration has not been tested. While the use of oxytetracycline aerosol spray in the treatment of burns is well known [40], our new oxytetracycline derivative polymeric films present the advantage of a more accurate dosage of the incorporated active substances, with superior effectiveness proven both by the nature of the incorporated derivatives and by means of the polymeric material, which also offers mechanical support, at the same time. 

## 5. Conclusions

New chitosan-derived oxytetracycline membranes were characterized with regard to structural and physico-chemical properties, swelling capacity and biodegradability and tested with respect to antimicrobial and antifungal activities. It has been found that all derivatives exhibited similar to slightly increased antimicrobial activity with parent oxytetracycline, while new chitosan membranes showed an improved swelling and biodegradation rate. An in vivo test proved that chitosan-derived oxytetracycline membranes showed also improved healing effects, which leads us to conclude that these new membranes could be useful in application as potential new dressings for burn wounds.

## Figures and Tables

**Table 1:** Target compounds, protonated precursors and fragment ions for oxytetracycline and derivate compounds.

Compound	Formula	Monitored Ion [MH]^+^	RT (min)	MS2 Fragments (m/z)
**OXI**	C_22_H_24_N_2_O_9_	461.15546	6.12	443.1448; 426.1183; 381.0605; 201.0546
**OXI-A**	C_29_H_27_N_3_O_11_	594.17183	8.65	426.1183; 201.0546; 381.0605; 151.0502 *
**OXI-B**	C_29_H_28_N_2_O_9_	549.18673	7.86	426.1183; 381.0604; 201.0546; **91.0542**
**OXI-C**	C_29_H_27_N_2_O_9_Br	627.09724	9.62	426.1183; 381.0604; 201.0546; **168.9647**
**OXI-D**	C_29_H_28_N_2_O_10_	565.18165	10.7	426.1183; 381.0604; 201.0546; **107.0491**

* In bold in table: the fragment ion characteristic of additional benzoic ring substituted with the specific radical.

**Table 2:** Physico-chemical properties of oxytetracycline derivatives.

Comp.	Chemical Formula	Theoretical Molecular Weight	M.P. (°C)	Ƞ (%)
OXI	C_22_H_24_N_2_O_9_	496.9	198	-
OXI-A	C_29_H_27_N_3_O_11_	630.02	176	76.80
OXI-B	C_29_H_28_N_2_O_9_	585.02	146	83.85
OXI-C	C_29_H_27_N_2_O_9_Br	663.92	127	69.23
OXI-D	C_29_H_28_N_2_O_11_	601.02	115	70.33

**Table 3:** Diameter of the inhibition area of the tested compounds (NT= not tested).

Tested Compound	Diameter of the Inhibition Area (mm)			
	*S. aureus* *ATCC 25923*	*E. coli* ATCC 25922	*P. aeruginosa* ATCC 27853	*C. albicans* ATCC 90028
OXI	33.16 ± 0.15	28.06 ± 0.15	18.16 ± 0.15	15.03 ± 0.15
OXI-A	25.2 ± 0.26	22.1 ± 0.1	12.03 ± 0.15	13.1 ± 0.1
OXI-B	33.1 ± 0.3	25.1 ± 0.1	20.11 ± 0.10	13.15 ± 0.15
OXI-C	31.2 ± 0.2	25.03 ± 0.15	17.06 ± 0.15	12.03 ± 0.15
OXI-D	31.23 ± 0.05	25.06 ± 0.05	19.1 ± 0.1	14.1 ± 0.2
Tetracycline 30 µg/disc	31.06 ± 0.15	25.16 ± 0.15	NT	NT
Ciprofloxacin 5 µg/disc	NT	34.1 ± 0.1	28.03 ± 0.15	NT
Fluconazole 25 µg/disc	NT	NT	NT	33.01 ± 0.2
Voriconazole 1 µg/disc	NT	NT	NT	30.15 ± 0.05
DMSO	0	0	0	0

**Table 4:** Minimum inhibitory concentration and minimum bactericidal concentrations of the tested compounds.

Tested Compounds	*S. aureus* ATCC 25923		*E. coli* ATCC 25922		*P. aeruginosa* ATCC 27853	
	MIC (mg/mL)	MBC (mg/mL)	MIC (mg/mL)	MBC (mg/mL)	MIC (mg/mL)	MBC (mg/mL)
OXI	0.009 0.0095 ± 0.0002	0.036 0.0362 ± 0.0003	0.036 0.0361 ± 0.0006	0.14 0.141 ± 0.003	0.073 0.0731 ± 0.0004	0.58 0.58 ± 0.005
OXI-A	0.036 0.0363 ± 0.0003	0.073 0.0734 ± 0.0003	0.073 0.0732 ± 0.0003	0.58 0.58 ± 0.004	0.29 0.291 ± 0.004	1,17 1.173 ± 0.02
OXI-B	0.009 0.0094 ± 0.0002	0.036 0.0363 ± 0.0005	0.036 0.0361 ± 0.0004	0.073 0.0733 ± 0.0006	0.073 0.0734 ± 0.0003	0.58 0.58 ± 0.003
OXI-C	0.009 0.0091 ± 0.0002	0.036 0.0361 ± 0.0003	0.073 0.0732 ± 0.0004	0.29 0.291 ± 0.004	0.14 0.141 ± 0.003	0.58 0.58 ± 0.006
OXI-D	0.009 0.0093 ± 0.0002	0.036 0.0362 ± 0.0005	0.036 0.0361 ± 0.0005	0.14 0.142 ± 0.003	0.073 0.0733 ± 0.0006	0.58 0.581 ± 0.003

Green: Antimicrobial activity comparable with oxytetracycline; Red: antimicrobial activity superior to oxytetracycline; Dark Red: antimicrobial activity inferior to oxytetracycline.

## Data Availability

No new data were created.
